# Nutrition and Liver Disease

**DOI:** 10.3390/nu10010009

**Published:** 2017-12-23

**Authors:** Claudia Mandato, Antonella Di Nuzzi, Pietro Vajro

**Affiliations:** 1 Pediatrics, Santobono - Pausilipon Pediatric Hospital, 80100 Naples, Italy; cla.mandato@gmail.com; 2 Department of Medicine, Surgery and Dentistry “Scuola Medica Salernitana”, Pediatrics Section, University of Salerno, 84081 Baronissi (Salerno), Italy; antonelladinuzzi@gmail.com

**Keywords:** nutrition, liver disease, cholestasis, liver transplantation, assessment, support

## Abstract

Malnutrition in children and adults with advanced liver disease represents a tremendous challenge as the nutritional problems are multifactorial. This Editorial comments the articles appearing in this special issue of *Nutrients*, “Nutrition and Liver disease” dealing with multiple diagnostic and therapeutic features that relate the outcomes of liver disease to nutrition. To improve quality of life and prevent nutrition-related medical complications, patients diagnosed with advanced liver disease should have their nutritional status promptly assessed and be supported by appropriate dietary interventions. Furthermore specific food supplements and/or restriction diets are often necessary for those with hepatic conditions associated with an underlying metabolic or nutritional or intestinal disease.

Despite advances in both the assessment and management of patients with liver disease, the provision of appropriate nutritional support for these patients is frequently lacking, an omission that has a large impact on clinical outcomes and quality of life. Except for elimination diets indicated for some hereditary or metabolic diseases that affect the liver, the general nutritional recommendations for most cases of compensated liver disease are analogous to the customary recommendations for a balanced diet. Malnutrition in children and adults with advanced liver disease, however, remains a tremendous challenge. The nutritional problems of these patients are multifactorial, and commonly include decreased intake due to anorexia, hypermetabolism, increased energy loss, and increased energy needs (Figure 1). The complicated underlying metabolic scenario is characterized by reduced glycogen stores, reduced protein synthesis, decreased branched-chain amino acid (BCAA)/aromatic amino acid (AAA) or BCCA/tyrosine (BT) ratios (3.5:1→1:1), and disturbances in fat metabolism during fasting.

In this special issue of *Nutrients*, which focuses on “Nutrition and Liver Disease,” a number of contributors have presented novel information and additional perspectives on multiple diagnostic and therapeutic features that relate the outcomes of patients with liver disease to nutrition.

## 1. Skeletal Muscle Mass

Skeletal muscle mass (SMM) is determined by the balance between protein synthesis and breakdown. Skeletal muscle loss (SML) is a major complication of liver cirrhosis (LC). In clinical settings, the assessment of skeletal muscle mass (SMM) to identifysevere SML (or sarcopenia) has long represented a very simple and objective bedside clinical measure of liver disease severity, because SML is associated with liver disease prognosis. It has now recently enhanced thanks to the support of various new imaging methods for assessing core SMM.

In this issue of *Nutrients*, the prognostic value of SMM was reported by studies in various categories of hepatic conditions, and the information has furthered our knowledge on the following issues:

- Strict interdependence exists between (1) the prevention of depletion of SMM,(2) the early diagnosis of and curative therapy for hepatocellular carcinoma (HCC),(3) the preservation of liver functional reserve, and (4) improved liver disease outcomes [1]. Age of male patients, female gender, a Child–Pugh score, and increased tumor size were significantly related to the SMM index, as measured at the third lumbar vertebra by transverse computed tomography (CT) imaging, which is a commonly used tool for HCC in the clinical setting. The results of the study by Imai et al. overall confirmed that sarcopenia negatively impacts the survival of patients with HCC, and is a valuable prognostic factor that might be impacted by liver functional reserve and the clinical stage of HCC.
- Therelationship between the loss of SMM, as assessed by bioelectric impedance analysis (BIA), and liver fibrosis, as measured by virtual-touch-quantification (VTQ) and acoustic-radiation-force-impulse elastography in patients with chronic liver disease (CLD), was reported by Nishikawa et al. [2]. Interestingly, BCAAs-to-tyrosine ratio (BTR) showed the second strongest correlation with the VTQ level, and was an independent predictor for decreased SMM index.
- BothBTR and SMM, as evaluated by BIA, were also confirmed to be reliable predictors of outcome in patients with liver diseases in another study, which found that increased values of BTR and SMM were associated with the resolution of chronic hepatitis C in patients treated with interferon-free direct-acting-antiviral therapy [3].
- However, all that glitters is not gold, and these studies have both strengths and limitations. The study by Imai et al. [1] was based on imaging techniques, and, although these assessments are objective and not affected by defects in hepatic synthesis or retention of NaCl and water, they are either expensive or involve radiation and cannot easily be repeated to monitor progress. The other twostudies [2,3] that used BIA, which is based on a two-component model of body composition (fat and fat-free mass), may have drawbacks. As recently emphasized by Amodio et al. [4] the validity of this technique is in fact critically dependent on assumptions relating to tissue density and hydration.

In future clinical and research settings, hand-grip dynamometry, which can provide measures for risk stratification for all-cause death, cardiovascular death, and cardiovascular disease in the general population [5], should be also implemented for the assessment of muscle strength. Indeed, for patients with cirrhosis, muscle strength, as assessed by hand-grip dynamometry, appears to be an easy-to-measure, sensitive, and specific marker for depletion of SMM [6] and ispositively correlated with total body protein [5,7].

## 2. Nutritional Assessment and Support

Another major issue concerns the fine line between the need for a hypercaloric diet rich in proteins and the risk of hepatic encephalopathy and hyperammonemia in sarcopenic, chronically malnourished patients with end-stage liver disease. The appropriate nutritional support of adult cirrhosis before and after liver transplantation was the focus of three articles in this issue.

- The first article [8] recommends the performance of an accurate multidisciplinary assessment of malnutrition in order to optimize nutritional support, especially for those patients with elevated risk for malnutrition because of the severity of their native liver disease. The recommended daily requirements for nutrients and energy intake should be achieved through oral intake, oral supplementation, and enteral nutrition. Parenteral nutrition (PN) should be used for moderately or severely malnourished patients with cirrhosis who cannot be fed orally or enterally, or if they have fasted longer than 72 h [9].
- The important and difficult issues concerned with the careful assessment of nutritional status of patients who are candidates for liver transplantation were also emphasized in the articles by Ahmed Hammad et al. [10] and Yang et al. [11]. The perioperative nutritional interventions, including the use of synbiotics, micronutrients, branched-chain amino acid (BCAA) supplements, and immunonutrients; fluid and electrolyte balance, the partial substitution of conventional fats with medium-chain triglycerides, and carefully monitored supplementation using fat soluble vitamins for cholestasis were reviewed for both adult and pediatric patients. Children with chronic liver disease are particularly vulnerable to malnutrition, which can compromise growth and brain development. They should benefit from early intervention provided by a multidisciplinary team. Yang et al. in particular have focused on the nutritional needs and support of children with chronic liver disease [11]; they revised the issues by providing conclusions that are consistent with the most recent guidelines of the European Society of Paediatric Gastroenterology Hepatology and Nutrition (ESPGHAN) [12].

## 3. Hepatopathies due to Inherited Metabolic/Genetic Defects

A number of inherited metabolic/genetic defects, including those that require (a) specific well established dietary restrictions (galactosemia, hereditary fructose intolerance, inborn errors of the urea cycle such as citrin deficiency and related metabolic pathways),(b) the addition of specific drugs (e.g., tyrosinemia; Wilson disease), or (c) special food supplements such as uncooked starch (e.g., glycogen storage diseases), represent a further challenge not only for pediatricians but also for adult hepatologists at the time of transition of care [13]. A dietician familiar with metabolic disorders is often needed on the team caring for these patients [14].

Dietary intervention is an evolving and increasingly used therapy also for the novel group of Congenital Disorders of Glycosylation (CDG). Glycosylation consists in the covalent binding ofan oligosaccharide chain to the polypeptide side chains of a glycoprotein. The carbohydrate consists of a simple sugar (e.g., glucose, galactose, mannose, and xylose), an amino sugar (e.g., *N*-acetylglucosamine or *N*-acetylgalactosamine), or an acidic sugar (e.g., sialic acid or *N*-acetylneuraminic acid). Monosaccharide supplements are being evaluated in trials for more and more subtypes of CDG, because monosaccharides have relatively high safety, especially compared to experimental drugs, and are easy to administer. The mechanism is still poorly understood, although in CDG-Ib, alimentary addition of mannose appear to bypass the defective step (conversion of fructose-6-phosphate to mannose-6-phosphate) by allowing for the formation of mannose-6-phosphate via the action of hexokinase [15]. In their article, Morava and her group presented an accurate overview of very recent data on the contributions of nutritional therapy by evaluating many of the different options for nutritional therapy that have somehow been associated with a positive effect on liver function in CDG [16]. The questions these authors, however, pose include whether or not these dietary interventions are sufficient and whether these dietary interventions are the most efficient therapies for CDG. They remind the reader that mannose therapy for patients with mannose phosphate isomerase (MPI)-CDG has not only been found to be possibly hazardous at higher doses, but unfortunately also cannot prevent progressive fibrotic liver disease in about one third of affected patients. In addition, recent experience with galactose therapy has been found beneficial for several CDGs [17,18,19] without, however, completely alleviating all clinical symptoms. While recognizing the progress this approach represents, they also suggest that future therapy for CDG therapy will most likely include administration of activated monosaccharides instead of single dietary sugars. The efficacy and toxicity of these novel drugs, however, remain to be investigated in human trials as well after careful preliminary evaluations in animal models are made.

## 4. Hepatopathies Driven by Primary Nutritional/Intestinal Tract Diseases

**Figure 2:**
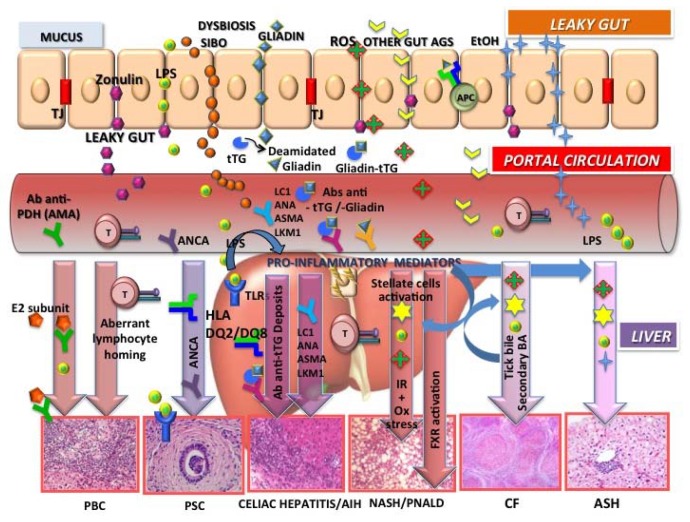
Gut-liver axis and liver diseases. Increased intestinal permeability and dysbiosis are common features linking the liver to a number of nutritional/gastrointestinal (GI) diseases depicted in the figure. The toll-like receptor (TLR)–bacterial lipopolysaccharide (LPS) interaction is one of the mechanisms involved in the release of proinflammatory mediators (cytokines), leading to liver inflammation and stellate-cell-activation-dependent fibrosis. Emerging therapeutic approaches that target the gut-liver axis therefore represent promising therapies to prevent or halt liver disease progression.

A number of liver diseases that are driven by primary nutritional/intestinal tract diseases (e.g., inflammatory bowel disease (IBD), celiac disease, cystic fibrosis (CF), alcoholic steatohepatitis (ASH), and non-alcoholic steatohepatitis (NASH) parenteral nutrition-associated liver disease(PNALD)), which are included under the umbrella term *gut-liver axisdysfunction* [20,21], also represent specific challenges. Investigations of the associations between the liver and diseased intestine are still preliminary, and further studies are essential for the clinical management of these complicated conditions (Figure 2).

Genetic predisposition and environmental stimulation determine the loss of tolerance in primary biliary cholangitis (PBC) where antimitochondrial antibodies (AMAs) cross-react with proteins from intestinal bacteria (E2 subunit), possibly because of molecular mimicry. Aberrant homing of intestinal lymphocytes also occurs. The association between celiac disease and PBC is also well established.

Closely associated with inflammatory bowel disease, primary sclerosing cholangitis (PSC) is also a heterogeneous disease that involves marked interactions between altered immune status (human leucocyte antigen (HLA)), altered bile composition, and host microbiome. Some subgroups of antineutrophil cytoplasmic antibodies (ANCAs) in the blood and bile are associated with increased liver enzyme levels and are biomarkers of liver disease with a possible role in the pathogenesis of the disease as well.

In celiac disease, immunoactive molecules generated from cross-linking between tissue transglutaminase and food/bacterial antigens reach the liver through the portal circulation and are responsible for transglutaminase antigen (tTG)–antibody complex hepatic deposits. The liver may respond to gluten either as a reactive “celiac hepatitis” or a true autoimmune hepatitis (with anti-liver cytosol (LC1), anti-nuclear antibodies (ANA), anti-smoot muscle antibodies (ASMA), anti-liver kidney microsomes (LKM1)) based on the DQ2 strong linkage disequilibrium with the DR3 and DR4, the latter being the major HLA risk factor for autoimmune liver disease.

Increased levels of bacterial endotoxin in the portal circulation suggest a role for gut-derived toxins also in the alcoholic liver diseasesteatohepatitis (ASH), non-alcoholic fatty liver disease (NAFLD/NASH), and parenteral nutrition liver disease (PNALD). Alcohol (EtOH) consumption and endogenous alcohol production by gut bacteria in obese individuals can disrupt the tight junctions (TJ) of the intestinal epithelial barrier, resulting in increased gut permeability. The bacterial endotoxin (lypopolisaccharides, LPS) contributes to inflammation through activation of toll-like receptor 4 (TLR4). Oxidative stress with reactive oxygen species (ROS), insulin resistance (IR), secondary bile acids (BAs), and farnesoid X receptor activation (FXR) represent major mechanisms of pathogenesis.

Animals with cystic fibrosis transmembrane conductance regulator defects and patients with cystic fibrosis have microbiomes that are different from individuals without CF, which might be accounted for by altered bile properties, prolonged small bowel transit, frequent antibiotic exposure, and small intestinal bacterial overgrowth (SIBO) (adapted and modified from [20]).

For celiac disease [22] and NASH [23,24], nutritional correction of the underlying gastrointestinal/nutritional disease may be effective in preventing the progression of liver disease. Readers are also referredto the 2017 *Nutrients* issue (ISSN 2072-6643) edited by Nobili and Alisi, where specific measures for the treatment of obesity-related NAFLD are specifically discussed.

Unfortunately, for some nutritional/intestinal conditions such as IBD and CF, the liver disease does not seem to be affected by decreases in the inflammatory activity in the intestinal tract [25] or improved function of the pancreas/respiratory tract [26].

Impaired intestinal function, directly or indirectly associated with gut dysbiosis, may also be one of the main pathogenic mechanisms of PNALD [27] (Figure 2).

- In this issue of *Nutrients*, Cahova et al. [28] reviewed studies of animal models and humans that suggested that chronic parenteral nutrition-related liver damage depends on intestinal failure and associated complications rather than PN administration per se. The prominent factors appear to be increased permeability of the intestinal barrier, which facilitates translocation of bacterial toxins and microorganisms into the portal circulation, mesenteric lymph nodes, and liver, and the overall proinflammatory status of the compromised intestine. The gut microbiota play a weighty role in the maintenance of the functional intestinal barrier and the establishment of either an immunotolerant or inflammatory intestinal setting [23,27]. Therapeutic strategies that focus on improving the composition of the microbiota through the targeted delivery of beneficial microbiota or by supplementation with immunomodulators are attractive areas of investigation.
- Alcoholic liver disease (ALD) is a strong predictor of malnutrition because of the numerous risk factors for malnutrition that are associated with both acute and chronic alcohol abuse. Due to complicated pathogenetic mechanisms, therapies for ALD and especially for severe alcoholic hepatitis (AH) are thorny problems in clinical practice. For severe acute AH, specific drug treatments, including glucorticoids and pentoxifylline, have been identified and currently are recommended by international guidelines. However, further elucidation of the mechanisms of pathogenesis is still needed [29]. In this context, the article by Xuchong Tang et al. [30] in this issue of *Nutrients* is particularly welcome. The study revealed that an artichoke extract exhibited significant preventive hepatoprotective effects not only for the carbon-tetrachloride-induced hepatoxicity [31,32] and NAFLD/NASH [33] as previously demonstrated but also against acute alcohol-induced liver injury (ASH). The effects probably depend on the ability of components of the extract not only to attenuate oxidative stress but also to suppress the toll-like receptor 4/nuclear factor kappa-light-chain-enhancer of activated Bcells (TLR4/NFkB) inflammatory pathway, a signaling pathway suggested to be one of the mechanisms of pathogenesis also of NAFLD through the overexpression of hepcidin [34].

## 5. Conclusions

In conclusion, to improve quality of life and prevent nutrition-related medical complications, patients diagnosed with advanced liver disease should have their nutritional status promptly assessed and be supported by appropriate dietary interventions. Furthermore, a dietary approach that uses specific food supplements and/or restriction diets is often necessary for patients with hepatic conditions associated with an underlying metabolic or nutritional or intestinal disease, due to the hepatoprotective and/or anti-oxidant and/or anti-inflammatory effects of these measures.

## Figures and Tables

**Figure 1:**
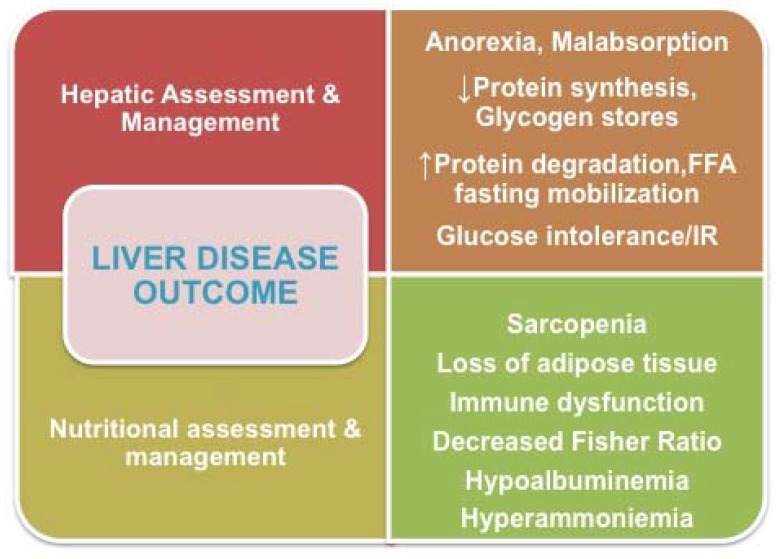
Diagnostic and therapeutic issues that link outcomes of liver diseases to nutrition.
